# The contribution of skeletal muscle interstitial cells to myogenesis

**DOI:** 10.1186/s13395-026-00414-9

**Published:** 2026-01-21

**Authors:** Fayez Issa, Frédéric Relaix, Joana Esteves de Lima

**Affiliations:** Univ Paris-Est Créteil, INSERM, U955 IMRB, F-94010, Créteil, France

**Keywords:** FAPs, Connective tissue, Myogenesis, Development, Regeneration

## Abstract

The developmental origin of skeletal muscle cell lineages has been extensively studied in the last 50 years. PAX3 + /PAX7 + myogenic progenitors are the foundation for skeletal muscle development and give rise to muscle satellite cells that support skeletal muscle postnatal growth, homeostasis and regeneration. Research into muscle connective tissue interstitial cells established their supportive role in muscle patterning during development and regeneration. With recent advances in mouse genetic lineage tracing, single cell transcriptomics and specific antibodies, the origin and the contribution of non-myogenic interstitial populations to the myogenic lineage have been reexamined. Growing evidence suggests that subpopulations of muscle interstitial cells actively contribute to myogenesis and fuse with myofibers during development, postnatal growth, and regeneration. In this review, we focus on the recent research advances that highlighted the direct contribution of interstitial cells, particularly connective tissue cells, to skeletal muscle growth and repair. We stress the importance of further investigating the mechanisms by which interstitial cells contribute to a myogenic fate in both physiological and pathological conditions.

## Introduction

Skeletal muscle is the most abundant tissue in the vertebrate body accounting for around 40% of total body mass. Although primarily recognized for its function in locomotion, skeletal muscle plays a significant role in metabolism and thermoregulation [67]. Skeletal muscle consists of multinucleated myofibers, each being formed of numerous repetitive monomeric units known as sarcomeres, which give skeletal muscle its characteristic striated appearance. The extensive exposure of skeletal muscle to external stress and stimuli is mitigated by its remarkable regeneration capacity, primarily driven by tissue-specific stem cells, the satellite cells. First identified in 1961, satellite cells were found in a specialized niche between the muscle fiber membrane (sarcolemma) and the surrounding basal lamina [39]. Satellite cells, characterized by the expression of the paired-homeobox transcription factor PAX7, exist in a dormant, quiescent, non-proliferative state under homeostatic conditions and ensure muscle growth during postnatal stages. However, upon muscle injury, satellite cells activate, proliferate, and undergo fate decision to either self-renew to sustain the stem cell pool or differentiate and fuse to regenerating fibers and restore skeletal muscle integrity.

Skeletal muscle is composed of distinct cell types beyond myofibers and satellite cells, the interaction of which is essential for maintaining tissue integrity. The interstitial cells constitute a large and heterogenous non-myogenic population that resides between the myofibers. In addition to immune cells, key interstitial cells include fibro-adipogenic progenitors (FAPs) (SCA1 + PDGFRα +), interstitial progenitor cells (PICs) (SCA1 + PW1/PEG3 +), smooth muscle mesenchymal cells (SMMCs) (ITGA7 + VCAM1 −), endothelial cells (ECs) (SCA1 + CD31 +), tenocytes (SCX +) and pericytes (NG2 + PDGFRβ +) [20, 24].

Within the interstitial cell pool there are mesenchymal stem cells (MSCs), multipotent stromal cells found in connective tissues of many organs including the skeletal muscle, known for their capacity to differentiate into fibroblasts, adipocytes and osteoblasts. MSCs play a crucial role in tissue repair and regeneration through the secretion of factors that allow crosstalk with other tissues. Although a subset of FAPs was shown to derive from embryonic OSR1 + MSCs, the origin of all FAP subpopulations in adults remains incompletely understood [64]. Within skeletal muscle, FAPs act as key regulators of muscle regeneration and homeostasis. FAPs were first identified as a distinct population of muscle-resident MSCs in 2010, by being isolated as a distinct cell population from adult mouse skeletal muscle based on their expression of PDGFRα and SCA1, while excluding hematopoietic, endothelial, and myogenic lineages [25, 61]. These studies established FAPs as non-myogenic, multipotent progenitors, capable of differentiating into fibroblasts and adipocytes, and highlighted their key role in orchestrating muscle regeneration after injury. In pathological conditions, such as heterotopic ossification, or when cultured in vitro in osteogenic-promoting conditions, FAPs possess chondrogenic and osteogenic potential [32, 65].

Pericytes are pluripotent mural vessel-associated cells that closely interact with endothelial cells to support and maintain capillary integrity. Beyond their supporting role, pericytes tightly regulate endothelial cell function and angiogenesis. An endothelial-adjacent TNAP + (tissue non-specific alkaline phosphatase) pericyte population has been identified as mesoangioblasts [8]. These cells share the expression of *Ng2*, MCAM (*Cd156*) and *Pdgfrb* with pericytes, but not all pericytes express TNAP. While mesoangioblasts contribute to myofiber formation in post-natal muscle growth, this is no longer the case in adult muscle [8]. During skeletal muscle development, pericytes originate from the paraxial mesoderm. During vascular development, endothelial cells secrete PDGFβ, which signals through PDGF receptor-β to recruit pericytes to the developing vessels [22]. Two distinct populations of pericytes were characterized in skeletal muscle, commonly referred to as type I and type II pericytes, based on the absence or presence of Nestin (NES) expression, respectively [5] (Table 1). In addition, while type I pericytes contribute to fibrogenesis in old mice, type II pericytes have myogenic capacity in vivo by fusing to regenerate myofibers [5]. Other interstitial cell populations include PW1 + progenitors. These cells were first identified in 2010 and are suggested to have myogenic potential both in vivo and in vitro [42] (Table 1).

**Table 1:** Summary of different interstitial cell populations' contribution to myogenesis

		Reference	Cell population	In vivo contribution	In vitro contribution
Chicken/Mouse	Embryo	Esteves De Lima et al., 2021 [15]	Fibroblasts	Contribution to myofibers at MTJ	Yes
Mouse	Embryo	Yaseen et al., 2021 [68]	Fibroblasts	Contribution to myofibers at MTJ	N/A
Mouse	Adult	Mitchell et al., 2010 [42] Pannérec et al., 2013 [49]	PEG3 + PAX7- cells (PICs)	Yes, following PICs injection into injured TA	Yes
Mouse	Adult	Liu et al., 2017 [35]	TWIST2 + cells (Tw2 +)	Yes, during muscle homeostasis and regeneration	Yes
Mouse	Adult	Flynn et al., 2023 [18]	HOXA11 + cells	Homeostasis Minimal contribution during regeneration	Yes
Mouse	Adult	Dellavalle et al., 2011 [10]	AP + pericytes	Homeostasis and regeneration	Yes
Mouse	Adult	Doyle et al., 2011 [11]	ABCG2 + pericytes	Minor contribution during regeneration	No
Mouse	Adult	Birbrair et al., 2013 [5]	NG2 + NES + pericytes	Yes, following injection into injured TA	Yes
Mouse	Adult	Mierzejewski et al., 2020 [41]	CD146 + NG2 + NES + pericytes	Yes, following subcutaneous transplantation	Yes
Mouse	Adult	Giordani et al., 2019 [20]	ITGA + VCAM1- SMMCs	Yes, following transplantation	Yes

*PEG3* paternally expressed gene 3, *PAX7* paired box 7, *TWIST2* twist basic helix-loop-helix transcription factor 2, *HOXA11* homeobox A11, *AP* alkaline phosphatase, *ABCG2* ATP binding cassette subfamily G member 2, *NG2* Neural/glial antigen 2, *NES* nestin, *CD146* cluster of differentiation 146, *ITGA7* lpha 7 integrin, *VCAM1* vascular cell adhesion molecule 1, *SMMCs* Smooth Muscle Mesenchymal Cells

While satellite cells were ultimately viewed as the sole contributors to regeneration and myofiber formation, recent studies highlighted the contribution of muscle-resident interstitial cells to myofiber formation during development, regeneration and homeostasis [18, 35]. In this review, we focus on the latest insights covering non-myogenic cell contribution to myogenesis and examine the potential underlying mechanisms and future perspectives.

### Connective tissue and skeletal muscle development

Myogenic development lays the foundation of muscle architecture during embryonic and fetal phases, where PAX3 +/PAX7 + myogenic progenitors sequentially commit into myoblasts and differentiate into myocytes, which ultimately fuse to form myofibers. This process is orchestrated by the myogenic regulatory factors, MYF5, MRF4, MYOD and MYOG, which control the progression from myoblast specification to terminal differentiation. During embryonic myogenesis, the primary fibers serve as structural scaffolds for subsequent muscle growth [30, 48]. In fetal stages, secondary myogenesis produces additional myofibers through fusion of fetal myocytes between themselves or with the primary fibers. This process is shaped by extrinsic signals, including patterning information derived from the surrounding connective tissue, and is dependent on innervation for proper development and survival of the secondary fibers [7, 51]. Fetal myogenesis includes the splitting and the growth of the muscle masses and the cell cycle exit of myogenic progenitors that acquire a satellite cell position under the fiber basal lamina [16, 50].

Proper myogenesis relies on a fine-tuned spatial interplay with connective tissue. While the drivers of limb development were broadly studied, the crosstalk between skeletal muscle and connective tissue is not well understood. In the limb, skeletal muscle derives from the somitic mesoderm, whereas muscle connective tissue cells originate from the lateral plate mesoderm. Work by Kardon and colleagues in the early 2000s, demonstrated that embryonic TCF7L2/TCF4 + connective tissue progenitors contribute to the formation of muscle connective tissue and muscle patterning [27]. More recent work on OSR1 + connective tissue cells demonstrated that OSR1-expressing progenitors in the embryonic limb mesenchyme are a key source of the adult FAP population and adipocyte lineages. At birth, around 25% of PDGFRα + cells originate from the OSR1 lineage. Subsequent lineage tracing analysis revealed that, like OSR1 + cells, TCF4 + embryonic mesenchymal progenitors give rise to adult FAPs. Although there is partial overlap, TCF4 and OSR1 mark largely distinct populations of embryonic muscle connective tissue cells [64].

During development, MSCs give rise to a wide range of connective tissues [52]. These are subdivided into distinct lineages defined by key transcription factors, including SCX + tendon progenitors [56], SOX9 + cartilage progenitors [3], RUNX2 + bone progenitors [12], and the irregular connective tissue that surrounds and patterns the muscle, marked by PDGFRα, OSR1 and TCF4 [27, 64]. The formation of these distinct tissues occurs temporally and spatially at the same time as myogenesis, and the interplay between the distinct cell populations contributes to their proper development.

### Connective tissue and muscle patterning

Irregular connective tissue provides a supportive and protective framework for various organs, ensuring mechanical strength and structural integrity. It is characterized by sparsely distributed cells embedded within a densely packed extracellular matrix. Over the past two decades, numerous studies showed that connective tissue plays a critical role in driving skeletal muscle patterning, a tightly regulated process by which muscle fibers are organized and arranged to form functional muscles with specific shapes, sizes, architectures, and attachment sites [26, 47]. During development, the dorsoventral patterning of the avian limb is determined by external signals from the overlying ectoderm resulting in the establishment of dorsal and ventral muscle masses. The dorsal ectoderm provides WNT signals such as WNT7A to specify dorsal muscle identity, while the ventral ectoderm provides BMP signals, like BMP4, to specify ventral muscle identity [13, 53]. This was demonstrated using classical embryo manipulation experiments by rotation of the limb ectoderm. Moreover, the partitioning of muscle masses and the tendon splitting depend on reciprocal interactions between the tissues, as demonstrated with the use of muscle-less and tendon-less avian hindlimb models [26]. The tendon-specific transcription factor Scleraxis (SCX) is broadly used as a driver for Cre expression in tendons. By crossing *Scx*^*CreERT2*^ and *Rosa*^*LSL−DTA*^ mice, where *Scx*-expressing cells are ablated following tamoxifen injection, it was demonstrated that the loss of tendon lineage in mouse embryos was accompanied by a defect in muscle patterning, shape and position. Particularly, while the SCX lineage is not required for the segregation of myoblasts within the limb bud or for early myofiber differentiation, it is indispensable for proper bundle morphology and attachment [47]. In the muscle, resident TCF4 + fibroblasts modulate the expression of the slow (MYH7) and fast myosin heavy chain IIb (MYH4) in fibers by secreted paracrine signals, both during development and in some muscles in the adult, as demonstrated by trans-well experiments [38]. Consistently, depletion of *Tcf4* in fibroblasts led to the decrease of *Myh7* and *Myh4* accompanied by an increase in the expression of embryonic myosin heavy chain (MYH3) during fetal myogenesis, indicating that repression of MYH3 depends on TCF4 expression in fibroblasts [38]. These results suggest that muscle connective tissue fibroblasts are crucial for skeletal muscle development. For a comprehensive overview of the role of connective tissue during muscle development, we suggest referring to the following review [45].

### Direct contribution of connective tissue cells to myogenesis during development

**Fig. 1:**
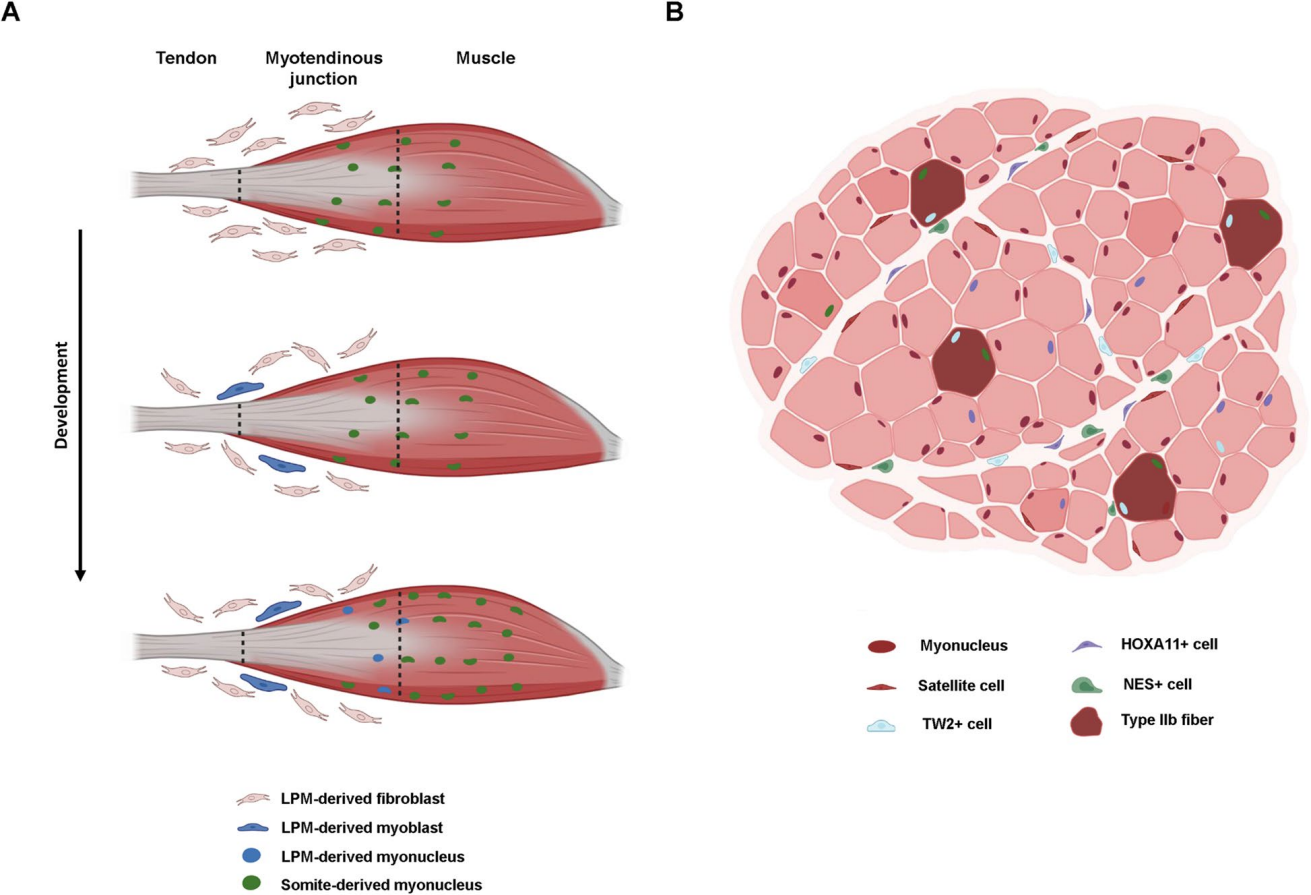
Contribution of fibroblasts to the myogenic lineage. **A** During development a subset of lateral plate-derived fibroblasts are capable of trans-differentiation into the myogenic lineage and fuse to developing fibers, particularly at the myotendinous junction.** B** Muscle interstitial cells including TW2 + (blue), HOAX11 + (purple) and NES + (green) cells fuse to myofibers in homeostasis and regeneration conditions. TW2 + and NES + interstitial cells primarily fuse to type IIb fibers

Although the role of the connective tissue during development was primarily assessed in the context of skeletal muscle patterning, recent evidence suggests that connective tissue-derived interstitial cells directly contribute to muscle fiber formation during development. With the use of a quail-into-chick presomitic mesoderm grafting approach, it was shown that around 10% of the MYOG + myocytes were not derived from the grafted presomitic-derived somites, which is the source of limb myogenic cells. The converse experiment, quail-into-chick lateral plate mesoderm grafts, showed that lateral plate mesoderm-derived nuclei were fused to muscle fibers and were preferentially located at the muscle tips, close to the myotendinous junction [15] (Fig. 1A,Table 1). PAX3 lineage tracing further confirmed these results, where analysis in mouse embryo forelimbs muscles at E15.5, showed that a subpopulation of PAX7 + and MYOD + cells were not derived from the PAX3 lineage. Conversely, genetic lineage tracing using the connective tissue markers SCX (tendon) and OSR1 (fibroblasts) showed that a subpopulation of myogenic cells (PAX7 +) was derived from these lineages [15]. Consistently, single-cell RNA sequencing (scRNAseq) analysis, performed on chick limb cells, identified a cell population with dual identity, expressing both myogenic (e.g., *PAX7, MYOD, MYOG*) and connective tissue markers (e.g., *PDGFRA, OSR1, SCX*). Further experiments demonstrated that BMP signaling drives the conversion of the lateral plate mesoderm-derived fibroblasts into PAX7 + myogenic cells, challenging the model that somites are the exclusive source of limb muscle cells [15]. A similar dual identity cell population that combined both skeletal muscle (*Pax7, Myf5*) and connective tissue (*Pdgfra*, *Osr1*) markers was identified at the myotendinous junction region in mouse newborn pups [68] (Fig. 1A,Table 1). This study revealed that the dual identity fibroblasts provided essential transcripts for neighboring myofibers, including the extracellular matrix (ECM)-modifying enzyme Lysyl oxidase-Like 3 (LOXL3), which is required for proper myofiber anchoring at the myotendinous junction [68]. By performing PRX1 lineage tracing (*Prx1*^*Cre/*+^), it was shown that the lateral plate mesoderm lineage gave rise to *Myod1*-expressing cells that fused with the developing fiber along the myotendinous junction (Fig. 1A). Transcripts associated with connective tissue identity, like *Loxl3* and *Pdgfra*, were found in fibers with fused lateral plate mesoderm-derived fibroblasts, suggesting that these cells trans-differentiated into myoblasts before fusing to myofibers [68].

Collectively, these results show that a subpopulation of lateral plate mesoderm-derived fibroblasts acquire a connective tissue-skeletal muscle double identity and contribute to myofiber formation as part of a physiological developmental process whose disruption alters myogenesis, particularly at the myotendinous junction.

### Interstitial cells contribution to myofiber formation

Skeletal muscle interstitial cells constitute a non-myogenic heterogenous population that resides between muscle fibers. Whether these cells have a myogenic potential is an active area of study. The primary role of interstitial cells as skeletal muscle supportive scaffold was extensively studied. However, a growing amount of evidence indicates that interstitial cell subpopulations contribute to myogenesis by fusing into myofibers. The idea that satellite cells are the only source of myonuclei in adult muscles was challenged by evidence in which interstitial cells contributed directly to myofiber formation and muscle repair [6, 10]. In this section, we discuss both the direct and indirect roles of muscle interstitial cells in adult muscle homeostasis and regenerative conditions.

### Supportive role of irregular connective tissue in adult muscle

FAPs and FAP-derived fibroblasts are the main cells composing the connective tissue of adult skeletal muscle [61]. They constitute 31% of mononucleated cells in resting muscle, around 20% at 5 days post injury (5dpi) and around 40% at 7 days post injury (7dpi) as shown by scRNAseq [9]. Numerous studies consistently highlighted FAPs’ role as supporting cells during skeletal muscle regeneration and various functions were identified. Firstly, FAPs contribute to muscle regeneration as the main ECM producer and remodeler. Some of the key ECM proteins secreted by FAPs include collagens, such as collagen I and collagen VI, which play a role in structural support and in the regulation of satellite cell function during homeostasis and regeneration [9, 63]. In addition, Periostin (POSTN, an ECM protein secreted by FAPs during regeneration, plays a critical role in modulating immune responses and ECM organization. Other FAP-secreted ECM molecules include Biglycan (BGN, required for proper ECM assembly, Fibrillin-1 (FBN1, important for elastic fiber assembly, Osteonectin (SPARC, which modulates cell–matrix interactions,and Fibronectin (FN1, crucial for cell adhesion and migration [46]. FAPs further contribute to ECM remodeling via the expression of regulatory enzymes such as lysyl oxidases and matrix metalloproteinases, which control collagen crosslinking and matrix turnover [62]. Although this coordinated and dynamic secretion of ECM molecules by FAPs is required for efficient muscle regeneration and functional tissue repair, the role of FAPs extends beyond structural support. FAPs actively secrete an array of cytokines and growth factors like IL-6, IL-10, and IL-33, WISP1, BMP1 and MMP14 during muscle regeneration. These factors are essential to orchestrate the cellular crosstalk that modulates the immune response and supports satellite cell function [33, 57, 58]. Collectively, FAPs integrate environmental signals and modulate regenerative processes, ensuring efficient muscle repair and preventing maladaptive processes such as fibrosis or fat deposition. Systemic ablation of PDGFRα + cells led to decreased immune cell and satellite cell numbers and fiber atrophy, in homeostasis and regeneration [66]. Another study demonstrated that the loss of a specific subtype of fibro-adipogenic progenitors (DPP4 + FAPs) led to a proportionate decrease in macrophage numbers in resting skeletal muscle [1]. These studies emphasize the pivotal role of FAPs in providing proper muscle signals under resting and regenerative conditions. For an extensive overview of FAP functions and interactions in muscle homeostasis and repair, we suggest referring to other literature reviews such as the following [4, 43, 46].

### Direct contribution of interstitial cells to myogenesis in adult muscle

Recent studies have challenged the overall premise that satellite cells are the sole contributors to myofiber formation during muscle growth, repair, and maintenance. Although satellite cells are essential and irreplaceable for myogenesis [55], accumulating evidence emphasizes the direct contribution of muscle interstitial cells to the myogenic lineage. Skeletal muscle homeostasis and repair are fundamentally supported by PAX7 + satellite cells, which reside between the sarcolemma and the basal lamina. Multiple studies confirmed that satellite cells are the principal self-renewing precursors essential for muscle fiber formation in postnatal stages [14, 47]. One of the earliest pieces of evidence of PAX7-negative progenitor cell contribution to myogenesis was presented in 2010 [42]. In this study, the authors identified a PAX7 negative muscle interstitial cell population (PICs) characterized by the expression of PEG3/PW1. Paternally expressed 3 (PEG3) is expressed in muscle progenitors and acts as a stress mediator via TNF-NFκB and p53 pathways [54]. When cultured in growth medium, 30% of PICs were positive for MYOD after 3 days and gave rise to myotubes under myogenic differentiation conditions. Cells that did not form myotubes were mostly positive for smooth muscle actin (αSMA) indicating that PICs constitute a bipotent progenitor cell population with both smooth and skeletal muscle potential [42]. Moreover, by *Pax3* lineage tracing, it was confirmed that PICs did not derive from PAX3 + cells. Grafting experiments of PICs into regenerating *tibialis anterior* (TA) muscles demonstrated a direct contribution of these cells to myofiber formation, one of the first examples of non-PAX3 cells contributing to myogenesis [42]. Follow-up studies on PEG3 + interstitial cells demonstrated the existence of two subpopulations based on PDGFRα expression [49] (Table 1). In vitro, the PEG3 + PDGFRα- population had myogenic potential, while PEG3 + PDGFRα + cells presented an adipogenic potential. When cultured in adipogenic conditions, the PEG3 + PDFGRα + showed an overlapping phenotype and expression profile with FAPs, suggesting a partial overlap between FAPs and PICs.

Another characterization of a skeletal muscle interstitial population was performed based on TWIST2 expression (Tw2 + cells) [35] (Table 1). The Tw2 + cells do not express PAX7 and show a distinct expression profile to that of satellite cells (PAX7 +). In homeostasis, using the *Tw2*^*CreERT2*^*;Rosa*^*tdTomato*^ lineage tracing mouse model, it was demonstrated that Tw2 + cells contributed to around 20% of total muscle fibers in the TA three weeks post-recombination, and to 60% of fibers two months post-recombination. This dynamic incorporation suggests that Tw2 + progenitors actively contribute to the physiological growth and maintenance of adult muscles [35]. Strikingly, the Tw2 + lineage only contributed to type IIb myofibers and its depletion led to the specific atrophy of type IIb fibers. During regeneration, the interstitial Tw2 + cells were observed in myofibers as early as 7 days post injury (7dpi), preferentially in type IIb (Fig. 1B). *TWIST2* expression is detected in human FAPs, as observed in scRNAseq data, suggesting that the Tw2 + progenitors exist in humans [28]. However, since most large mammals, including humans, lack the MYH4 protein (type IIb myofiber) [23], the functional role for the contribution of Tw2 + cells to human myofibers remains to be elucidated. As for their transcriptional identity, Tw2 + cells strongly express *Pdgfra*, *Sca1*, *Prrx1* and many collagen subtypes, suggesting a strong overlap with FAPs transcriptional profile [35]. Although negative for PAX7 in vivo, when cultured in myogenic growth medium, Tw2 + cells downregulate the expression of *Twist2* and commit to the myogenic lineage by upregulating *Pax7* and *Myod1* expression [35]. In addition, Tw2 + cells fused into myofibers in culture, as efficiently as PAX7 + cells, which exceeds the myogenic capacity of previously identified interstitial non-myogenic precursors.

It was recently demonstrated that a population of HOXA11-expressing interstitial cells contribute to myofiber formation during postnatal growth, but not in development [18] (Table 1). This suggests that the described HOXA11 + cells are likely distinct from the dual-identity fibroblasts previously characterized during skeletal muscle development [15, 68]. Lineage tracing for HOXA11 (*Hoxa11*^*iTom*^) at eight weeks of age and following single tamoxifen administration, revealed that HOXA11 lineage contribution to myofibers was variable between muscles and ranged from 100% of fibers in *extensor carpi ulnaris*, to 50% in *flexor digitorum profundus*, and 20% in the *flexor digitorum sublimis* muscles. In contrast, PAX7 + satellite cells contributed to only around 20% of fibers across all three muscles at eight weeks [18]. Long-term lineage tracing of HOXA11 + cells revealed contribution across all myofiber types, in contrast to Tw2 + cells, which contributed preferentially to type IIb myofibers [35] (Fig. 1B). While their fiber type contributions differ, immunostaining and scRNAseq indicate a substantial overlap between the two populations [18]. Indeed, published and available sc/snRNAseq datasets show that *Twist2*, *Hoxa11* and *Peg3* are expressed in subpopulations of FAPs both in resting and regenerating conditions [17, 21].

PDGFRα + cells were repeatedly shown to lack intrinsic myogenic potential in vitro [25, 62]. In contrast, Tw2 + cells efficiently formed myotubes when cultured alone in low serum medium [35]. The transcriptional profile of the Tw2 + population is consistent with previously characterized lateral plate mesoderm-derived FAPs, as evidenced by robust expression of ECM and stromal markers including *Sca1*, *Pdgfra*, *Col1a1*, *Fn1*, *Tnc*, *Prrx1* and *Postn*, among others [35]. This suggests that Tw2 + cells mark a PDGFRα + mesenchymal subpopulation with myogenic capacity. Although PDGFRα + cells did not show myogenic capacity [25, 62], one could speculate that manipulating the entire PDGFRα + cell population could hinder the potential myogenic capacity of Tw2 + cells. However, one cannot exclude possible inefficient construct design or non-specific lineage tracing. In homeostasis, FAPs can be subdivided into at least six transcriptionally distinct populations when analyzed bioinformatically, highlighting high levels of heterogeneity [36]. However, whether these subpopulations constitute independent populations in the adult muscle tissue remains understudied,while some developmental studies have begun to address this question [64]. Whether Tw2 + and HOXA11 + cells represent distinct or overlapping populations remains unclear. However, conversely to Tw2 + cells, HOXA11 + cells contributed to existing myotubes in vitro but failed to form *de-novo* myotubes, indicating that they lack intrinsic myogenic potential [18]. Moreover, in vivo, Tw2 + cells contributed solely to type IIb fast-twitch myofibers while HOXA11 cells contributed to all fiber subtypes (Fig. 1B). Therefore, even though Tw2 + and HOXA11 + interstitial cells show partial transcriptional overlap, they likely represent phenotypically and functionally distinct populations. One limitation of the above-mentioned studies is that Cre recombination was induced at early postnatal stages. The residual developmental plasticity of progenitors during early postnatal stages decreases as the tissues mature. Accordingly, the plasticity present at the time of Cre induction may impact lineage outcomes. In addition, in the case of the HOXA11 study, the lineage tracing was performed using the *Rosa*^*LSL−H2BmCherry*^ mouse line where the reporter is nuclear, consistent with the idea that Tomato signal observed in myofibers is due to direct nuclear accretion from HOX11 + cells. However, it was shown that Tomato signal can be transmitted to myofibers via secreted extracellular vesicles from the surrounding cells, in the absence of fusion [44]. Moreover, independent studies showed the existence of myonuclear protein movement within a single myofiber [37, 60]. Therefore, the contribution of non-myogenic cells to myofibers based solely on lineage tracing experiments should be interpreted with caution.

Satellite cells are the main actors of skeletal muscle regeneration, and satellite cell depletion is accompanied by major regeneration defects [34, 55]. Distinct research groups showed that muscle hypertrophy can occur in the absence of satellite cells [19, 29, 40]. Moreover, it was demonstrated that hypertrophy occurs by the activation of protein synthesis by myonuclei, independently of satellite cell fusion. One could speculate that hypertrophy occurs via myofiber accretion in which Tw2 + or HOXA11 + populations could contribute to such processes. Strikingly, comparing Hoxa11iTom and Pax7iTom lineage tracing models in two-month-old animals showed more Hoxa11 lineage contribution to myofibers compared to the Pax7 lineage. However, this observation contradicts the fact that there is no compensation in the number of myonuclei in satellite cell-depleted mice [34], suggesting a lack of contribution from other cell types to myonuclei addition. One must also take in consideration that in the case of Hoxa11 lineage tracing, the contribution to myofibers was observed in a time-restricted manner at fetal and early postnatal time-points. Functional experiments showed that conditional depletion of Tw2 + cells induced a type IIb fiber atrophy in mice, while no conditional ablation of the Hoxa11 + lineage has been documented [35]. The exact mechanisms by which non-myogenic cells trans-differentiate and fuse into myofibers remains unknown. Whether these cells completely or partially retain their original identity before fusing or transition through a common PAX7 + state prior to nuclear accretion remains unclear. However, BMP signaling was shown to mediate the conversion of a subset of fibroblasts to myoblasts during chicken embryo development [15]. It is therefore crucial to unify the nomenclature of the distinct subpopulations of fibroblasts and FAPs capable of trans-differentiation into myoblasts and uncover the molecular mechanisms that drive the activation of the myogenic program in the specific subsets of fibroblasts/PDGFRα-expressing cells. In the embryo, the localization of fibroblast-derived myonuclei preferentially at the myotendinous junction, supports the existence of a spatially restricted signal that could guide the fibroblast-to-myoblast conversion. Consistently, it was shown that some fibroblast transcripts are crucial for proper myotendinous junction development [31]. In parallel, conditional depletion of PDGFRα + cells during muscle regeneration is associated with a delay in regeneration and smaller myofiber cross-sectional area [66]. This phenotype could reflect the altered crosstalk between FAPs, satellite cells and immune cells. A scRNAseq experiment on adult resting skeletal muscle identified a previously overlooked interstitial ITGA7 + VCAM1 − cell population that was designed as smooth muscle mesenchymal cells (SMMCs) [20] (Table 1). These cells showed contribution to myofibers following transplantation but to a much lower extent than satellite cells [20].

### Pericyte contribution to myogenesis

Pericytes are mesenchymal vascular-associated progenitor cells. Human skeletal muscle pericytes, marked by the expression of NG2 proteoglycan and alkaline phosphatase, were shown to possess myogenic capacity when co-cultured with satellite cells, where half the pericytes fused into myotubes [10] (Table 1). Surprisingly, when cultured alone, around one third of pericytes spontaneously differentiated into myocytes (*Myod1* and *Myog* expression) and formed myotubes [10]. In vivo experiments, with an intra-femoral artery systemic delivery of pericytes, showed a direct contribution to myofibers (*TNAP*^*CreERT2*^*;Rosa*^*LacZ*^) of immunodeficient dystrophic mice, ten-fold greater than that observed with satellite cells transplantation [10]. Unlike Tw2 + and HOXA11 + cells, pericytes also contributed to the satellite cell pool, where one month after tamoxifen induction, approximately one in four satellite cells in the *pectoralis* muscle and one in ten in the triceps muscle derived from pericytes [10] (Fig. 1B). However, when satellite cells are ablated in *Pax7*^*DTR*^ mice, the satellite cell pool is not rescued by other cell types [34, 55].

A pericyte (SCA1 + NG2 + CD31 +) subpopulation characterized by the expression of *Abcg2* was identified in 2011 [11] (Table 1). Phenotypically, these cells showed considerable overlap with endothelial cells and pericytes. Lineage tracing of ABCG2 (*Abcg2*^*CreERT2*^*;Rosa*^*LacZ*^) showed that these cells present a minor contribution to myogenesis through direct fusion with regenerating myofibers. Interestingly, ABCG2-derived cells did not contribute to myofibers in the mdx model of chronic muscle degeneration, which suggests context-dependent restriction to contribute to myofiber formation [11]. Despite the direct contribution of ABCG2 + cells to myofibers, the primary role of these cells in regeneration remains myofiber-indirect, through their differentiation into vascular-associated interstitial cells and immune microenvironment modulation.

Further studies classified pericytes into two groups based on Nestin (NES) expression: type I and type II pericytes in the absence or presence of NES expression, respectively [5]. Functionally, only the type I pericytes (NG2 + NES-) give rise to fibroblasts in vitro. Conversely, only the type II pericytes (NG2 + NES +) actively fused to form myofibers in low serum medium. When transplanted into injured TA muscles, type II pericytes had a higher contribution to myofibers in young versus old mice, showing that the aging microenvironment limits their regenerative capacity. Like Tw2 + cells, type II pericytes contributed exclusively to fast type II fibers in the *soleus* muscle (Fig. 1B). This similarity in type II-specific fiber fusion observed in pericytes (NG2 + NES +) and Tw2 + cells suggests a shared mechanism in the contribution to myofibers. It is possible that they transition through a common intermediate state before preferentially fusing into type II fibers. A more recent study showed the contribution of pericytes to myogenesis by observing positive Tomato signal in myofibers using the NG2Cre mouse line crossed with the *Rosa*^*Tomato*^ [59]. Of note, the number of Tomato + fibers was higher in the slow twitch muscle *soleus* compared to *gastrocnemius* and conditional ablation of these cells was accompanied by a strong atrophy particularly in the soleus [59].

A distinct interstitial progenitor population within skeletal muscle was identified based on CD146 expression [41] (Table 1). CD146 + cells express both pericyte markers NG2 and NES suggesting that CD146 marks the type II pericyte cells [5]. These cells were able to fuse with regenerating myofibers in vitro and in vivo. However, their fusion efficiency remained lower than that of satellite cells. These studies demonstrated that NES + but not NES- pericytes contribute to myogenesis in the adult skeletal muscle.

## Conclusion and perspectives

Skeletal muscle is a heterogenous tissue composed of myofibers, satellite cells and a diverse array of interstitial cells, essential for tissue maintenance. The supportive role of interstitial cells in skeletal muscle homeostasis and repair has been thoroughly described. However, evidence for the contribution of interstitial non-myogenic cells to myofiber formation continues to emerge. In this review, we brought together recent work that challenges the long-standing consensus that somite-derived, PAX7-expressing cells are the sole contributors to myogenesis in developmental and adult contexts. However, this question requires careful discussion, taking into consideration possible experimental caveats. Moreover, the validation of these processes requires additional, functional experiments, such as specific deletion of contributing cells, to address the impact on myofiber formation. In addition, results of lineage tracing experiments should be interpreted cautiously since one cannot rule out the possibility that the interstitial cell markers used in each study are expressed at some stage during the myogenic differentiation. In fact, recent findings demonstrated that during muscle regeneration, a subpopulation of myogenic cells express *Scx*, a transcription factor identified as a tendon progenitor-specific marker [2].

The extent to which interstitial populations contribute to the myogenic lineage and to different fiber types in adult skeletal muscle remains controversial. The need for deeper mechanistic understanding of these processes is crucial for our understanding of muscle development, homeostasis, and regeneration. In a neuromuscular disease context, such as Duchenne Muscular Dystrophy (DMD), the satellite cell pool is exhausted due to chronic fiber damage and inflammation. Shifting the focus towards other interstitial cell types with myogenic potential emerges as a promising therapeutic strategy. Consequently, strategies designed to activate the dormant myogenic program in cells such as pericytes and FAPs require further investigation as a potential approach to mitigate satellite cell depletion and enhance muscle repair in neuromuscular disorders.

## Data Availability

No datasets were generated or analysed during the current study.
